# Sepsis mortality among patients with haematological malignancy admitted to intensive care 2000–2022: a binational cohort study

**DOI:** 10.1186/s13054-024-04932-0

**Published:** 2024-05-06

**Authors:** Aleece MacPhail, Claire Dendle, Monica Slavin, Robert Weinkove, Michael Bailey, David Pilcher, Zoe McQuilten

**Affiliations:** 1 School of Public Health and Preventive Medicine, Faculty of Medicine, Nursing and Health Sciences, Monash University, 553 St Kilda Road, Melbourne, VIC 3004 Australia; 2 Department of Infectious Diseases, Monash Health, 246 Clayton Road, Clayton, VIC 3168 Australia; 3 School of Clinical Sciences, Faculty of Medicine, Nursing and Health Sciences, Monash University, 246 Clayton Road, Clayton, VIC 3004 Australia; 4 Department of Infectious Diseases, Peter MacCallum Cancer Centre, 305 Grattan Street, Melbourne, VIC 3000 Australia; 5 National Centre for Infections in Cancer, Sir Peter MacCallum Department of Oncology, University of Melbourne, 305 Grattan Street, Melbourne, VIC 3000 Australia; 6 Cancer Immunotherapy Programme, Malaghan Institute of Medical Research, Wellington, New Zealand; 7 Te Rerenga Ora Wellington Blood and Cancer Centre, Wellington Hospital, Te Whatu Ora Health New Zealand Capital Coast & Hutt Valley, Wellington, 6021 New Zealand; 8 Department of Intensive Care, Alfred Health, 55 Commercial Road, Prahran, VIC 3004 Australia; 9 Australian and New Zealand Intensive Care Society Centre for Outcome and Resource Evaluation (ANZICS-CORE), 101 High St Prahran, Victoria, 3001 Australia; 10 The Australian and New Zealand Intensive Care Research Centre (ANZIC-RC), School of Public Health and Preventative Medicine, Monash University, 553 St Kilda Rd, Prahran, VIC 3004 Australia; 11 Department of Haematology, Monash Health, 246 Clayton Road, Clayton, VIC 3168 Australia

## Abstract

**Background:**

Sepsis occurs in 12–27% of patients with haematological malignancy within a year of diagnosis. Sepsis mortality has improved in non-cancer patients in the last two decades, but longitudinal trends in patients with haematological malignancy are not well characterised. We aimed to compare outcomes, including temporal changes, in patients with and without a haematological malignancy admitted to ICU with a primary diagnosis of sepsis in Australia and New Zealand over the past two decades.

**Methods:**

We performed a retrospective cohort study of 282,627 patients with a primary intensive care unit (ICU) admission diagnosis of sepsis including 17,313 patients with haematological malignancy, admitted to 216 intensive care units (ICUs) in Australia or New Zealand between January 2000 and December 2022. Annual crude and adjusted in-hospital mortality were reported. Risk factors for in-hospital mortality were determined using a mixed methods logistic regression model and were used to calculate annual changes in mortality.

**Results:**

In-hospital sepsis mortality decreased in patients with haematological malignancy, from 55.6% (95% CI 46.5–64.6%) in 2000 to 23.1% (95% CI 20.8–25.5%) in 2021. In patients without haematological malignancy mortality decreased from 33.1% (95% CI 31.3–35.1%) to 14.4% (95% CI 13.8–14.8%). This decrease remained significant after adjusting for mortality predictors including age, SOFA score and comorbidities, as estimated by adjusted annual odds of in-hospital death. The reduction in odds of death was of greater magnitude in patients with haematological malignancy than those without (OR 0.954, 95% CI 0.947–0.961 vs. OR 0.968, 95% CI 0.966–0.971, *p* < 0.001). However, absolute risk of in-hospital mortality remained higher in patients with haematological malignancy. Older age, higher SOFA score, presence of comorbidities, and mechanical ventilation were associated with increased mortality. Leukopenia (white cell count < 1.0 × 10^9^ cells/L) was not associated with increased mortality in patients with haematological malignancy (*p* = 0.60).

**Conclusions:**

Sepsis mortality has improved in patients with haematological malignancy admitted to ICU. However, mortality remains higher in patients with haematological malignancy than those without.

**Graphical abstract:**

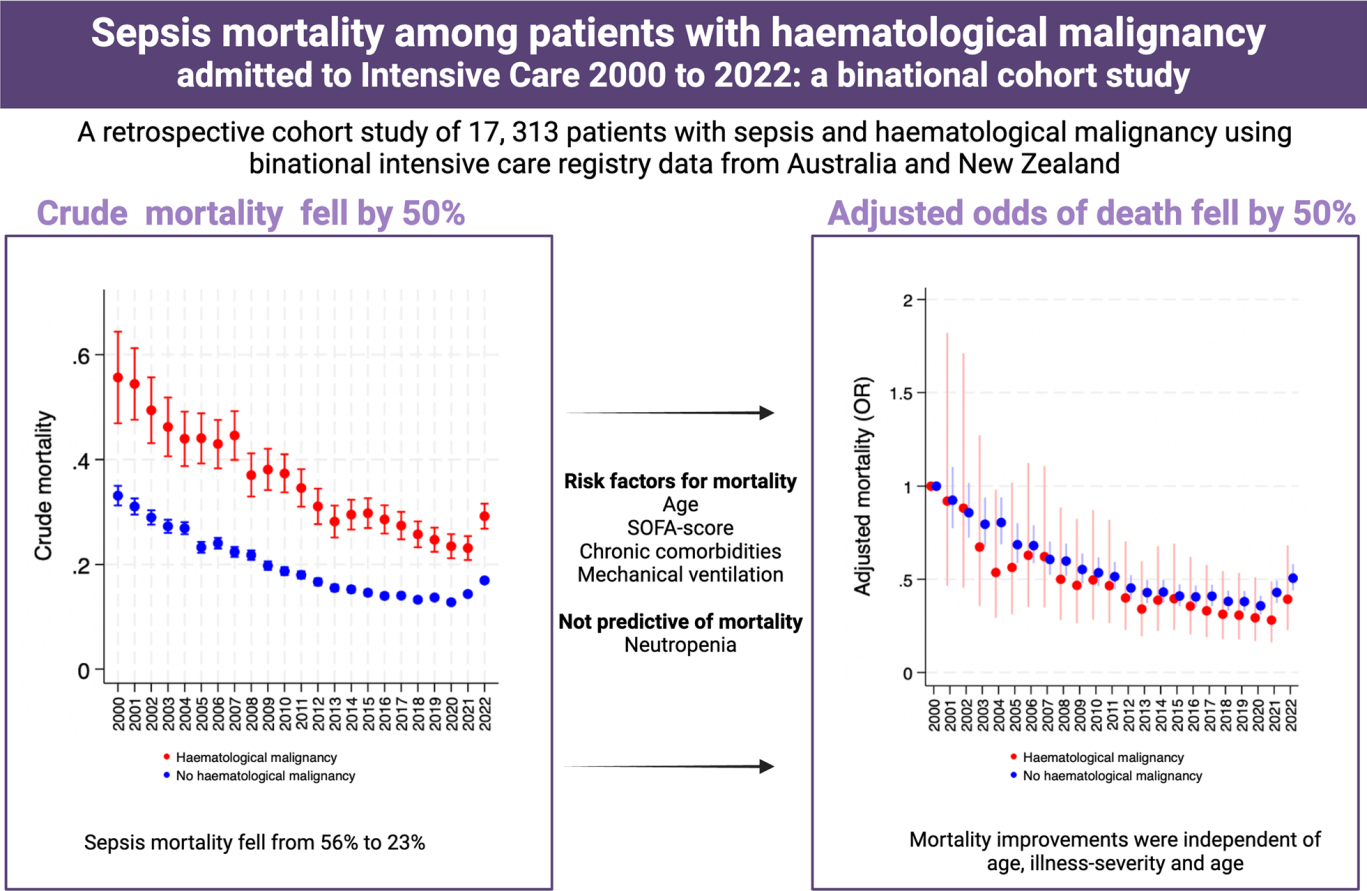

**Supplementary Information:**

The online version contains supplementary material available at 10.1186/s13054-024-04932-0.

## Key points

Among patients with haematological malignancy and sepsis admitted to intensive care, mortality has fallen by over 50% in the last two decades.

Improvements are independent of organ failure, neutropenia, and age; however, mortality remains higher than for patients with no malignancy.

**Supplementary Information:**

The online version contains supplementary material available at 10.1186/s13054-024-04932-0.

## Introduction

Sepsis is a life-threatening complication of infection, characterised by dysregulated host response and organ dysfunction. Sepsis has a profound impact on the care of patients with haematological malignancy. Both haematological malignancies and their treatments predispose to infection, and sepsis is reported in 12–27% of patients with haematological malignancy within the first year of treatment [1]. Admission to an Intensive Care Unit (ICU) is required in up to 15% [2], and short term mortality is reported at 30–60% [3, 4]. Among those who recover, consequences extend well beyond the initial episode, due to delays to life-saving cancer therapy, prolonged antimicrobial exposure, and colonisation with multi-resistant organisms [5]. In addition, hospital length of stay is tripled and healthcare costs are increased [1]. A 2021 Canadian study estimated excess healthcare costs of $CAD 46,154 in the first 12 months of sepsis for patients with haematological malignancy [6]. In the United States, sepsis accounts for 15% of all cancer-related hospitalisation costs, or $3.4 billion in 2004 [1].

Sepsis outcomes have improved over the past two decades. For example, between 2000 and 2012 in Australia and New Zealand, mortality in patients with sepsis admitted to ICU fell from 35 to 17%, a relative risk reduction of 47.5% [7]. Similar findings have been reported in the United States and Europe [8, 9]. Changes in sepsis outcomes among those with haematological malignancies have not been well-defined during this time period, which has seen new treatments for haematological malignancies resulting in improved disease-specific survival [10], and a shift in demographics of those treated for haematological malignancies, with a growing cohort of older patients and those with comorbidities [10, 11].

As recently as the late 1990s and early 2000s admission of patients with haematological malignancy to the ICU was considered controversial [12] based on very poor survival, particularly for those requiring mechanical ventilation [13]. Relevant ICU outcome data are critical to inform rational decision-making about treatment escalation or de-escalation, and to counsel patients and relatives. Single centre studies from the mid 2000s onward suggest survival in septic patients with cancer admitted to ICU [14, 15] may have improved. However, contemporary data describing outcomes of patients with sepsis and haematological malignancy remain limited. We investigated a large, binational registry of ICU admissions and outcomes in from 2000 to 2022, and reported sepsis outcomes and longitudinal trends among patients with and without an underlying haematological malignancy.

## Objectives

To compare outcomes, including temporal changes, in patients with and without a haematological malignancy admitted to ICU with a primary diagnosis of sepsis in Australia and New Zealand over the past two decades.

## Methods

### Study design

Retrospective cohort study of all patients admitted to ICU in Australia or New Zealand between January 2000 and December 2022, with a primary ICU admission diagnosis of sepsis.

### Ethical approval

The study was approved by the Alfred Hospital human research ethics committee, Melbourne, Australia, with a waiver of informed consent (Project Number 292/20).

### Setting

Data were extracted from the Australian and New Zealand Intensive Care Society Adult Patient Database (ANZICS-APD). The ANZICS-APD is an electronic database that collects episodes of care from ICUs in Australia and New Zealand, including patient demographics, primary diagnoses, comorbidities, physiological data from the first 24 h of admission, mortality and length of stay. The ANZICS-APD presently receives data on approximately 190,000 ICU admissions each year from 216 centres, representing 98% of all adult ICU admissions in Australia and 67% in New Zealand [16].

### Inclusion criteria

All patients aged 16 years or older, admitted to ICUs in Australia or New Zealand between January 2000 and December 2022, with a primary ICU admission diagnosis consistent with sepsis were included.

Only first admissions to ICU were included, readmission episodes were excluded. Patients discharged to another ICU were excluded to avoid duplication. Patients were excluded if they were admitted to ICU for palliative care or organ donation, and if no hospital mortality data were recorded.

### Definitions

Sepsis was defined as either (1) documented primary admission diagnosis of sepsis or (2) primary admission diagnosis of infection and organ dysfunction measured by Sequential Organ Failure Assessment (SOFA) score of ≥ 2, as defined by the Third International Consensus Definitions for Sepsis and Septic Shock (Sepsis-3) [17]. Primary ICU admission diagnoses were coded according to the ANZICS modification of the Acute Physiological and Chronic Health Evaluation (APACHE) diagnostic coding system [16]. Only one admission diagnosis was recorded.

Comorbid diagnoses of leukaemia or myeloma (grouped together) or lymphoma were recorded for calculation of the APACHE scores. Haematological malignancy was defined as comorbid diagnosis of leukaemia/myeloma and/or lymphoma. Leukopenia was defined as white cell count (WCC) < 1.0 × 10^9^ cells/L.

Illness severity scores reported were the APACHE II/III/IV score, Australian New Zealand Risk of Death (ANZROD), and SOFA score, and were calculated as previously described [18–20]. ANZROD is a locally derived and highly discriminatory mortality prediction model used for benchmarking ICU outcomes and is valid for ICU admissions from 2012 onwards. Hospital mortality was defined as death prior to discharge or transfer to another facility.

Hospital classification was defined as tertiary (a large teaching hospital), metropolitan (non-tertiary hospital in a large town), rural/regional (small non-teaching hospital located outside large cities/towns) or private. Hospital remoteness classifications were based on the Australian Department of Health Modified Monash Model. [21].

### Statistical analysis

Descriptive analysis of patient demographics, clinical characteristics and outcomes was performed. Results were reported as n (%), mean (standard deviation) and median (interquartile range) as appropriate. For dichotomous variables, means with 95% confidence intervals (CI) calculated using binomial proportion were reported. Group comparisons were made using Chi-square tests for equal proportion, students t-test or ANOVA for normally distributed outcomes, and Wilcoxon-Mann–Whitney or Kruskal–Wallis tests otherwise.

A logistic regression model was fitted to identify independent predictors of hospital mortality for patients with sepsis. Variables included in the model were chosen based on association with mortality previously described in the literature [22, 23] and include age; sex; presence of comorbid cardiovascular, respiratory, liver or renal disease; presence of leukopenia; SOFA score; presence of a treatment limitation order; planned admission to ICU; post-operative status; hospital classification type (tertiary, metropolitan, rural or private); hospital and ICU admission source; mechanical ventilation; year of admission and interactions between the above. In the final model, only variables that were consistently collected across the study period were included. To avoid collinearity with variables of interest (i.e. diagnosis of haematological malignancy, leukopenia, sepsis diagnosis), the final model used SOFA score as the chosen indicator of illness severity, fitted as a categorical variable in quartiles. A mixed effects model was used with patients nested within sites and site treated as a random effect.

Variables independently associated with hospital mortality were used to calculate adjusted odds of death over time. Adjusted odds of death over time were compared between patients with and without haematological malignancy. To compare the change in outcome over time between patients with haematological malignancy and those without, an interaction term between haematological malignancy and year of admission was also fitted with year of admission treated as a continuous variable. Year was fitted firstly as a categorical variable to establish linearity, and then secondly as a continuous variable to facilitate a measure of annual decline. Sensitivity analysis was performed using sites that contributed data across the entire study period, and for the period 2010–2019. Subgroup analysis of patients requiring mechanical ventilation and those with more than two organ failures was performed.

All logistic regression results have been reported as odds ratios (ORs) and 95% CIs. Linearity between each continuous variable and the dependent variable was demonstrated. In case of nonlinearity, the variable was stratified based on inflection points and clinical significance. For categorical variables with multiple levels, the reference level was attributed to the one with the lowest probability of the dependent variable. Analyses were performed using Stata version 18.0 (Stata Corporation, College Station, TX, USA).

## Results

### Clinical characteristics

We identified 317,422 ICU admissions for patients aged ≥ 16 years in Australia or New Zealand with a primary diagnosis of sepsis during the study period. Of these patients, 34,795 were excluded (21,895 readmissions, 10,279 transfers to another ICU, 887 admitted for palliative care and 1,734 with missing mortality data) leaving 282,627 patients included in the final analysis (Additional file 1: Supplementary material, Fig. S1). Of these, 17,313 (6.1%) had a haematological malignancy.

Data completeness was > 99% for sex, age, SOFA, APACHE and ANZROD scores; mechanical ventilation and ICU LOS, > 98% for hospital length of stay (LOS), and 92% for white cell count. Treatment limitation data was consistently collected from 2008 onward (> 85% completeness over this period).

Clinical characteristics of sepsis admissions with and without haematological malignancy are reported in Table 1. Among those with WCC data collected, leukopenia was present in 30.0% of patients with haematological malignancy, compared to 1.6% in patients without haematological malignancy. Patients with haematological malignancy were more likely to be male, less likely to have one or more chronic comorbidities, more likely to have a limitation of treatment order in place, more likely to be admitted to a tertiary hospital and less likely to have a planned admission or a post-operative infection than those without haematological malignancy. Patients with sepsis and haematological malignancy were more likely to be admitted from the ward than patients without haematological malignancy, were more likely to have high illness severity scores, including APACHE-II and SOFA, but less likely to receive mechanical ventilation (Table 1).

**Table 1:** Clinical characteristics in sepsis in patients with and without haematological malignancy

	No haematological malignancy (n = 265,314)		Haematological malignancy (n = 17,313)	
	n	(%)	n	(%)
Male sex	147,615	(55.7%)	10,987	(63.5%)
Age median (IQR)	66.7	(53.2, 76.8)	66.1	(56.3, 74.3)
*Comorbidities*				
Cardiovascular disease	25,800	(9.7%)	911	(5.3%)
Respiratory disease	27,296	(10.3%)	796	(4.6%)
Liver disease	7467	(2.8%)	174	(1.0%)
Renal disease	16,137	(6.1%)	597	(3.4%)
> / = 1 of the above	61,918	(23.3%)	2044	(11.8%)
Treatment limitation*^a^	29,710	(13.3%)	2401	(16.4%)
Leukopenic^a^	3834	(1.6%)	4783	(30.1%)
Mechanical ventilation	79,480	(30.0%)	3957	(22.9%)
*Risk stratification*				
SOFA score, median (IQR)^a^	4	(3, 7)	6	(4, 8)
APACHE II score, median (IQR)^a^	18	(13, 24)	22	(18, 28)
ANZROD predicted risk of death, median (IQR)^a^	0.08	(0.03, 0.24)	0.22	(0.09, 0.50)
ANZROD score, mean (SD)	0.18	(0.22)	0.32	(0.28)
Planned admission to ICU	10,157	(9.3%)	362	(4.9%)
Post-operative infection	26,418	(10.0%)	391	(2.3%)
*Infection source*				
Urinary	37,664	(14.20)	1003	(5.79)
Skin and soft tissue	37,664	(3.49)	151	(0.87)
Respiratory	37,664	(24.15)	3239	(18.71)
Abdominal/GI	19,610	(7.39)	280	(1.62)
Other	6815	(2.57)	152	(0.88)
Not recorded	127,892	(48.20)	12,488	(72.13)
*Hospital classification*				
Rural	51,938	(19.6%)	1860	(10.7%)
Metropolitan	69,637	(26.2%)	2919	(16.9%)
Tertiary	115,473	(43.5%)	10,154	(58.6%)
Private	28,266	(10.7%)	2380	(13.7%)
*ICU admission source*				
OT/recovery	27,868	(10.5%)	482	(2.8%)
ED	126,940	(47.9%)	6222	(36.0%)
Ward	75,608	(28.5%)	9297	(53.7%)
Other**	34,688	(13.1%)	1302	(7.5%)

All comparisons were statistically significant (*p* < 0.001)

*IQR* interquartile range, *SOFA* sequential organ failure assessment, *APACHE* acute physiology and chronic health score, *ANZROD* Australia New Zealand risk of death ICU intensive care unit, *LOS* length of stay

^*^Treatment limitation: data routinely collected from 2008

^**^Other includes direct admission to ICU e.g. from Hospital in the Home; transfer from external ICU or external hospital; transfer from internal ICU

^a^Unknown data excluded from analysis

SOFA quartiles were in patients with sepsis and haematological malignancy were as follows: Q1 = SOFA < 4; Q2 = SOFA 4–5; Q3 = SOFA 6–7; Q4 = SOFA 8–21.

### Outcomes

Mortality over the study period was higher in patients with haematological malignancy than patients without haematological malignancy (hospital mortality 31.2% vs. 17.0%; ICU mortality 20.5% vs. 11.3%; *p* < 0.0001). This difference remained significant after adjusting for age, illness severity, presence of comorbidities, post-operative status, and year of admission (OR 2.29; 95% CI 2.02–2.60, *p* < 0.001). Patients with haematological malignancy had lower likelihood of discharge home, and survivors with haematological malignancy had significantly longer hospital length of stay (Table 2).

**Table 2:** Outcomes in sepsis in patients with and without haematological malignancy

	No haematological malignancy (n = 265,314)		Haematological malignancy (n = 17,313)		*p*
	n	(%)	n	(%)	
*Outcome*					
Death in hospital	45,218	(17.0%)	5405	(31.2%)	< 0.001
Death in ICU^a^	29,846	(11.3%)	3537	(20.5%)	
Discharged home	167,348	(63.1%)	9643	(55.7%)	
Discharged to rehabilitation or long-term care facility	20,563	(7.8%)	885	(5.1%)	
*LOS in survivors*					
LOS hospital days, median (IQR)^a^	10.9	(6.2, 20.7)	14.9	(7.7, 29.0)	< 0.001
LOS ICU days, median (IQR)^a^	2.8	(1.6, 5.4)	2.8	(1.6, 5.1)	< 0.02

*IQR* interquartile range, *ICU* intensive care unit, *LOS* length of stay

^a^Unknown data excluded from analysis

### Risk factors for mortality in patients with and without haematological malignancy

Independent risk factors for mortality identified in multivariable analysis for patients with sepsis, with and without haematological malignancy, are shown in Table 3. Older age, higher SOFA score, presence of comorbidities, and mechanical ventilation were all associated with increased mortality.

**Table 3:** Independent risk factors for hospital mortality in patients with sepsis with and without haematological malignancy

	Univariable			Multivariable		
	OR	(95% CI)	*p*	OR	(95% CI)	*p*
Male sex	0.87	(0.86–0.89)	< 0.001	1.02	(1.00–1.05)	0.06
Age	1.03	(1.03–1.03)	< 0.001	1.03	(1.03–1.03)	< 0.001
>/= 1 chronic comorbidity*	1.70	(1.66–1.73)	< 0.001	1.63	(1.59–1.67)	< 0.001
*SOFA score quartile*			0.001			< 0.001
Q1 (reference)	–	–		–	–	
Q2	1.71	(1.66–1.77)		1.51	(1.45–1. 56)	
Q3	2.80	(2.71–2.90)		2.27	(2.19–2.36)	
Q4	7.31	(7.10–1.53)		5.59	(5.39–5.79)	
Post-operative status	0.32	(0.30–0.33)		0.34	(0.32–0.36)	
*Hospital classification*						
*Rural (reference)*						
Metropolitan	1.16	(1.12–1.19)	< 0.001	1.00	(0.90–1.12)	0.90
Tertiary	1.37	(1.34–1.41)	< 0.001	1.16	(1.04–1.29)	0.01
Private	1.04	(1.00–1.08)	0.03	0.98	(0.88–1.09)	0.71
Mechanical ventilation	2.61	(2.55–2.66)	< 0.001	1.94	(1.89–2.00)	< 0.001
Haematological malignancy	2.26	(2.17–2.36)	< 0.001	2.29	(2.02–2.60)	< 0.001
*Leukopenia*						
Leukopenia*no HM	3.92	(3.67–4.18)	< 0.001	3.04	(2.83–1.27)	< 0.001
Leukopenia*HM	2.37	(2.23–2.52)	< 0.001	0.98	(0.90–1.06)	0.60
*Year of admission*						
Year of admission*HM	0.997	(0.994–1.00)	0.02	0.954	(0.947–0.961)	< 0.001
Year of admission*no HM	0.950	(0.949–0.952)	< 0.001	0.968	(0.966–0.971)	< 0.001

*SOFA* sequential organ failure assessment, *HM* haematological malignancy, *OR* odds ratio, *CI* confidence interval

^*^Chronic comorbidities = chronic cardiovascular, respiratory, renal, or liver disease

Comparisons between haematological malignancy and no haematological malignancy were assessed using interaction terms between haematological malignancy and each variable of interest. Model AUROC = 0.78

Leukopenia was independently associated with increased mortality in patients without haematological malignancy (*p* < 0.001), but not in patients with haematological malignancy (*p* = 0.60).

### Change in outcomes from 2000 to 2022

**Fig. 1:**
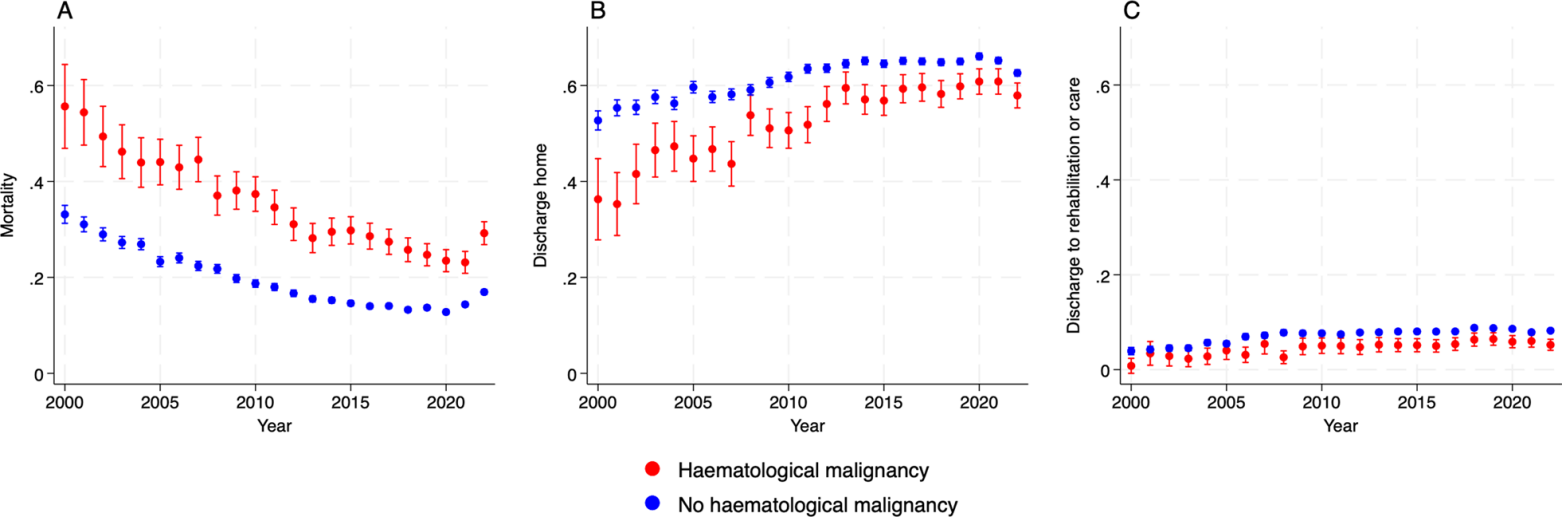
Crude likelihood of hospital outcome in patient with and without haematological malignancy. **A** crude mortality **B** discharge home **C** discharge to rehabilitation or long-term care. Error bars indicate 95% confidence interval

Crude in-hospital sepsis mortality decreased significantly in patients admitted to ICU with and without haematological malignancy over the study period (Fig. 1). In patients with haematological malignancy, mortality fell from 55.6% (95% CI 46.5–64.6%) in 2000 to 23.1% (95% CI 20.8–25.5%) in 2021. In patients without haematological malignancy, mortality fell from 33.1% (95% CI 31.3–35.1%) to 14.4% (95% CI 13.8–14.8%). Both groups were more likely to be discharged home, and more likely to be discharged to rehabilitation or care (Fig. 1).

The decrease in mortality over the study period was greatest in young patients < 45 years (Additional file 1: Supplementary material, Fig. S2), and was observed across all SOFA score quartiles (Additional file 1: Supplementary material, Fig. S3).

There was a slight but statistically significant increase in mortality in patients without haematological malignancy in 2021, and in both groups in 2022, coinciding with the Omicron surge in COVID-19 cases in Australia.

**Fig. 2:**
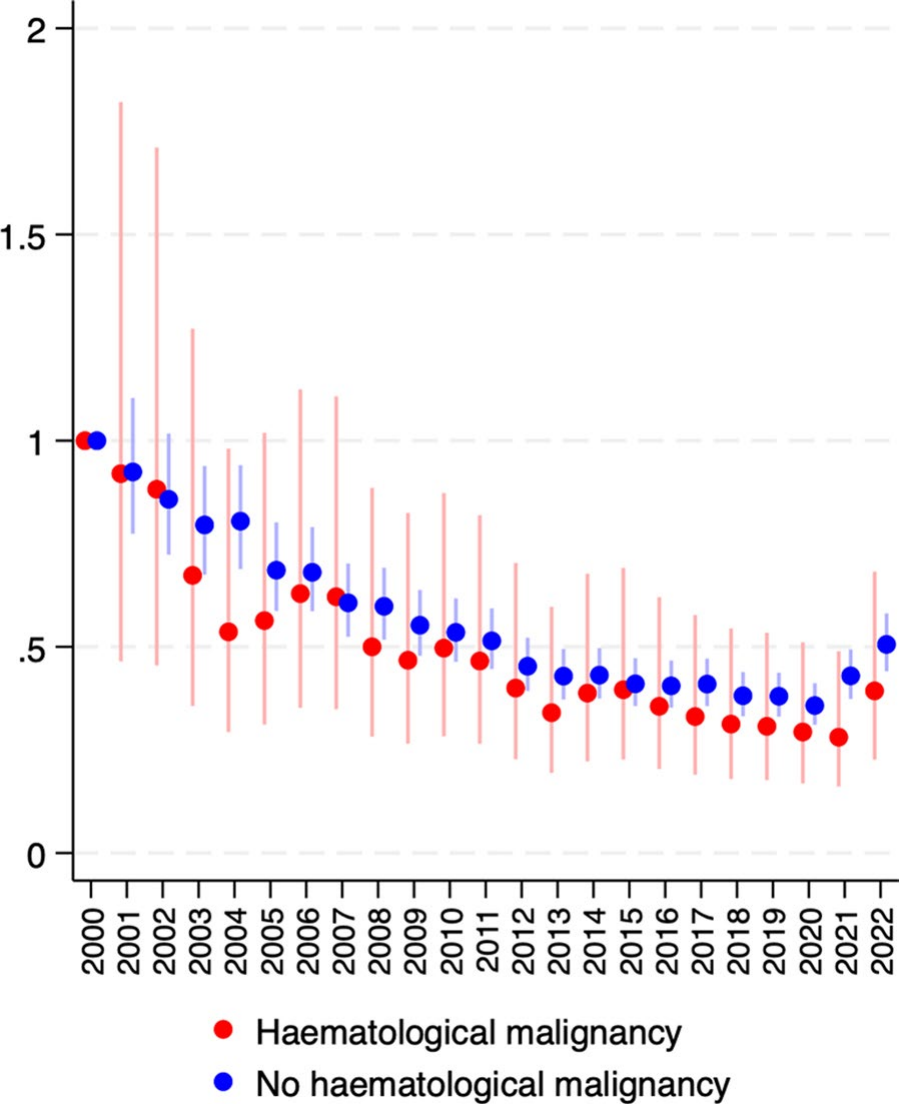
Adjusted risk of mortality by year in patients with and without haematological malignancy. Results displayed as odds ratio ± 95% confidence interval relative to the year 2000. Adjustments made for age, sex, presence of >/= 1 chronic comorbidity, SOFA score (quartile), post-operative status, mechanical ventilation, leukopenia and hospital site as a random effect

After adjustment for predictors of mortality including age, SOFA score, ventilation status, and leukopenia, the reduction in observed mortality remained significant. Adjusted sepsis mortality declined over the study period in patients with and without haematological malignancy (Fig. 2). The odds of death for patients with haematological malignancy declined by 4.6% per year over the 23 year period (OR 0.954 95% CI 0.947–0.961). For patients without haematological malignancy odds of death declined by 3.2% per year (OR 0.968, 95% CI 0.966–0.970). The interaction term between haematological malignancy and year of admission was significant, indicating this reduction was greater in patients with haematological malignancy than those without haematological malignancy (*p* < 0.001). The reduction in adjusted odds of mortality was associated with an increase in the adjusted odds of discharge home (Additional file 1: Supplementary Fig. S4).

### Mechanically ventilated patients

Within the subset of ICU sepsis admissions with haematological malignancy who were mechanically ventilated (n = 3,957; 22.8% of all haematological malignancy sepsis admissions), mortality trends were similar to unventilated patients. Crude mortality declined from 73% in 2000 (95% CI 59–84%) to 53% in 2021 (95% CI 45–61%); *p* = 0.01 (Additional file 1: Supplementary material, Fig. S5.) After adjustment for predictors of mortality including older age, SOFA score, and leukopenia, this reduction in mortality remained significant (OR death for year of admission = 0.963, 95% CI 0.951–0.976, *p* < 0.001, Additional file 1: Supplementary material, Fig. S6).

### Organ failures

Number of organ failures is reported in Additional file 1: Fig. S7. Among patients with haematological malignancy and sepsis, 119/17,313 (0.69%) had more than two organ failures. This group had very high mortality (87% over the study period). Likelihood of more than two organ failures decreased over the study period (OR per year 0.95, 95% CI 0.92–0.7, *p* < 0.001). Annual mortality reduced modestly over the study period 94.9% (95% CI 82.7%–99.4%) in 2000–2009 versus 83.8% (95% CI 73.8–91.1%) in 2010–2022 (pooled due to low numbers).

### Sensitivity analysis

To exclude the possibility that the observed temporal changes were due to changes in the ICUs contributing cases to the ANZICS-APD, we conducted a sensitivity analysis incorporating only sites that contributed data throughout the full study period. This included 58/216 sites (26%) and 129,508/153,118 patients (54%). The reduction in odds of death by year for patients with haematological malignancy and sepsis remained significant in sensitivity analysis (OR 0.958, 95% CI 0.950–0.966, *p* < 0.001).

A second sensitivity analysis including the period 2010–2019 was performed, based on the observation that mortality improvements were most marked in the first decade of the study period and increased during COVID-19 pandemic conditions. Reductions in mortality remained statistically significant during this period for patients with haematological malignancy and sepsis (OR death per year compared to 2010 0.96, 95% CI 0.939–0.984, *p* = 0.001), but not for those with haematological malignancy and sepsis who were mechanically ventilated (OR 0.97, 95% CI 0.93–1.02, *p* = 0.21, Additional file 1: Supplementary Fig. S8).

## Discussion

We analysed 23 years of binational data describing patients with sepsis admitted to ICUs in Australia and New Zealand. Over the study period, sepsis mortality declined for all patients, with a greater reduction among those with an underlying haematological malignancy compared to those without. Sepsis mortality in haematological malignancy patients fell substantially, including among those with higher illness severity and those who were mechanically ventilated.

Despite this, in hospital mortality among patients with sepsis admitted to ICU remains worse in patients with a haematological malignancy compared to those without (mortality of approximately 23% vs. 14% in 2021). In addition, improvements in mortality have plateaued in the last decade, and we observed an increase in mortality across all patient groups in 2022, corresponding with the peak of SARS-CoV-2 admissions in Australia. Finally, the subgroup of patients with more than two organ failures, while representing < 1% of all admissions, had very poor prognosis with mortality over 80%.

Our findings extend the existing literature describing improved sepsis outcomes in other patient groups. Kaukonen and colleagues reported an overall improvement in sepsis outcomes in Australia and New Zealand between 2000 and 2012 [7], although did not specifically report outcomes for patients with haematological malignancy. Lemiale and colleagues described outcomes among 2062 patients in seven European ICUs from 1994 to 2015 with a mix of solid tumour and haematological malignancies, and found year of admission to be an independent predictor of 30 days survival [24]. Zampieri and colleagues performed a longitudinal analysis in Brazil of patients with cancer, including solid tumours, and with infective or non-infective critical illness; they reported a reduction in overall mortality between 2011 and 2019. However, very poor prognosis in the subset of patients with haematological malignancy and more than two organ failures has also been recently reported in a Dutch retrospective cohort study [25]. To our knowledge, our study is the largest and most current study specifically describing sepsis in patients with haematological malignancy admitted to intensive care.

The impact of the SARS-CoV-2 pandemic on non-SARS-CoV-2 mortality has also been previously described and may relate to increased strain on hospital and ICU systems [26]. A large population based study in the United States described increased non-SARS-CoV-2 mortality in 2020–2021, with the greatest impact in hospitals with highest prevalence of SARS-CoV-2 [27]. In Australia and New Zealand, SARS-CoV-2 cases were at their highest point in late 2021 to 2022, having remained low until the point in the setting of prolonged stay-at-home orders [28].

Higher SOFA score, older age, and requirement for mechanical ventilation were all associated with increased risk of mortality among patients admitted to ICU for sepsis. Leukopenia (leukocytes < 1.00 × 10^9^ cells/L) was independently associated with mortality for patients without haematological malignancy, but was not a risk factor among patients with haematological malignancy. Previous studies describing the impact of neutropenia on sepsis mortality have yielded conflicting results, possibly related to heterogeneity of included patients [29] However, a population based cohort study of 43,466 patients with cancer and septic shock in Korea reported lower mortality in those with neutropenia [30]. We used leukopenia of < 1.00 × 10^9^ cells/L as a surrogate for neutropenia, and observed a significant interaction between the effect of leukopenia and the presence of haematological malignancy. In those with haematological malignancy, neutropenia was not independently associated with mortality. Our findings add to a body of literature suggesting neutropenia is not a major predictor of outcome for patients with haematological malignancy and sepsis.

Improvements in mortality reported in this study may be related to improvements in treatment of infection, supportive care, or cancer therapy, among other mechanisms. It is possible that changing patterns of patient selection for admission to ICU also contributed to improved outcomes. However, reductions in mortality were stable after adjustment for mortality predictors including illness severity, and were consistent across SOFA quartiles, suggesting that these findings are not explained by admission practices alone. Moreover, attitudes toward ICU admission of patients with haematological malignancy was historically more restrictive than they are today [12].

Our findings highlight the importance of using contemporaneous outcome data to guide treatment escalation decisions, and when counselling patients and relatives of those admitted to ICU with sepsis. Improved sepsis outcomes, including the subset of patients requiring mechanical ventilation and those with neutropenia, suggest that critically ill haematology malignancy patients are increasingly likely to benefit from ICU care. Our findings support early referral to ICU in patients with haematological malignancies and suspected or confirmed sepsis, particularly as rapid escalation of care has been associated with reduced mortality [31]. Among those with haematological malignancies, leukopenia is not a predictor of worse outcome. Conversely, our findings emphasise the clinical importance of non-neutropenic sepsis, which accounted for a majority of sepsis cases and was associated with similar outcomes. Although outcomes have improved over time, mortality remains higher for patients with haematological malignancy. Given the evolving landscape of therapies for haematology patients, resultant immunosuppression and patient characteristics (who are increasingly older and more comorbid), further research into sepsis prevention, treatment, and post-sepsis care are required. Finally, the specific impacts of the SARS-CoV-2 pandemic on patients with malignancy and critical illness including sepsis warrants further investigation.

Our study has several limitations. We included all patients with a primary diagnosis of sepsis based on Sepsis-3 criteria and/or ANZICS-APD primary clinical diagnosis of sepsis. The approach may under-represent clinical sepsis, particularly in patients admitted with multiple medical complications, and does not capture those who develop sepsis after being admitted to ICU for another reason. The grouping of all patients with haematological malignancy is also a limitation. Patients with haematological malignancy are clinically diverse, and we did not have access to details of primary diagnosis, disease status at admission, treatment history, or stem cell transplant recipient status. Strengths of the study include the cohort size, study duration and use of a binational registry with high levels of data completeness, and including 216 ICUs, which increases the generalisability of our findings. To our knowledge, this is the largest longitudinal cohort study of sepsis outcomes in patients with haematological malignancies.

The impact of specific haematological malignancy diagnoses and treatments, the absence of neutropenia, and the presence of SARS-CoV-2 on sepsis outcomes are areas for future research. Studies that extend critical care registry data with other clinical and microbiological data will be important in expanding these findings.

## Conclusion

Over the past two decades, there have been substantial improvements in mortality in haematological malignancy patients with sepsis, including across all levels of illness severity and in patients requiring mechanical ventilation. However, mortality and post-ICU length of stay remain higher than other patients with sepsis, and further research into prevention and treatment in patients with haematological malignancies is warranted.

## Supplementary Information

**Additional file 1**. Supplementary material.

## Declarations

### Data availability

Access to information from ANZICS CORE is restricted due to confidentiality requirements pertaining to the use of clinical quality registry data in Australia and New Zealand. Specific requests may be considered on a case by case basis. Enquiries can be made to anzics.core@anzics.com.au.

## References

[1] Williams MD, Braun LA, Cooper LM, Johnston J, Weiss RV, Qualy RL, et al. Hospitalized cancer patients with severe sepsis: analysis of incidence, mortality, and associated costs of care. Crit Care. 2004;8(5):1–8. 15469571 10.1186/cc2893 PMC1065011

[2] Schellongowski P, Staudinger T, Kundi M, Laczika K, Locker GJ, Bojic A, et al. Prognostic factors for intensive care unit admission, intensive care outcome, and post-intensive care survival in patients with de novo acute myeloid leukemia: a single center experience. Haematologica. 2011;96(2):231. 21071501 10.3324/haematol.2010.031583 PMC3031690

[3] Manjappachar NK, Cuenca JA, Ramírez CM, Hernandez M, Martin P, Reyes MP, et al. Outcomes and predictors of 28-day mortality in patients with hematologic malignancies and septic shock defined by sepsis-3 criteria. J Natl Compr Cancer Netw. 2022;20(1):45–53. 10.6004/jnccn.2021.7046 34991066

[4] Nazer L, Lopez-Olivo MA, Cuenca JA, Awad W, Brown AR, Abusara A, Sirimaturos M, Hicklen RS, Nates JL. All-cause mortality in cancer patients treated for sepsis in intensive care units: a systematic review and meta-analysis. Support Care Cancer. 2022;30(12):10099–109. 36214879 10.1007/s00520-022-07392-w PMC9549043

[5] Winters BD, Eberlein M, Leung J, Needham DM, Pronovost PJ, Sevransky JE. Long-term mortality and quality of life in sepsis: a systematic review. Crit Care Med. 2010;38(5):1276–83. 20308885 10.1097/CCM.0b013e3181d8cc1d

[6] Tew M, Dalziel K, Thursky K, Krahn M, Abrahamyan L, Morris AM, et al. Excess cost of care associated with sepsis in cancer patients: results from a population-based case-control matched cohort. PLoS ONE. 2021;16(8):e0255107. 34379649 10.1371/journal.pone.0255107 PMC8357157

[7] Kaukonen K-M, Bailey M, Suzuki S, Pilcher D, Bellomo R. Mortality related to severe sepsis and septic shock among critically Ill patients in Australia and New Zealand, 2000–2012. JAMA. 2014;311(13):1308–16. 24638143 10.1001/jama.2014.2637

[8] Law AC, Stevens JP, Walkey AJ. National trends in timing of death among patients with septic shock, 1994–2014. Crit Care Med. 2019;47(11):1493–6. 31397713 10.1097/CCM.0000000000003956 PMC9893726

[9] Lorencio Cárdenas C, Yébenes JC, Vela E, Clèries M, Sirvent JM, Fuster-Bertolín C, et al. Trends in mortality in septic patients according to the different organ failure during 15 years. Crit Care. 2022;26(1):302. 36192781 10.1186/s13054-022-04176-w PMC9528124

[10] Sant M, Minicozzi P, Mounier M, Anderson LA, Brenner H, Holleczek B, et al. Survival for haematological malignancies in Europe between 1997 and 2008 by region and age: results of EUROCARE-5, a population-based study. Lancet Oncol. 2014;15(9):931–42. 25030467 10.1016/S1470-2045(14)70282-7

[11] Kumar SK, Dispenzieri A, Lacy MQ, Gertz MA, Buadi FK, Pandey S, et al. Continued improvement in survival in multiple myeloma: changes in early mortality and outcomes in older patients. Leukemia. 2014;28(5):1122–8. 24157580 10.1038/leu.2013.313 PMC4000285

[12] Groeger JS, Aurora RN. Intensive care, mechanical ventilation, dialysis, and cardiopulmonary resuscitation: implications for the patient with cancer. Crit Care Clin. 2001;17(3):791–803. 11525058 10.1016/s0749-0704(05)70208-6

[13] Rosolem MM, Rabello LS, Lisboa T, Caruso P, Costa RT, Leal JV, et al. Critically ill patients with cancer and sepsis: clinical course and prognostic factors. J Crit Care. 2012;27(3):301–7. 21855281 10.1016/j.jcrc.2011.06.014

[14] Legrand M, Max A, Peigne V, Mariotte E, Canet E, Debrumetz A, et al. Survival in neutropenic patients with severe sepsis or septic shock. Crit Care Med. 2012;40(1):43–9. 21926615 10.1097/CCM.0b013e31822b50c2

[15] Pène F, Percheron S, Lemiale V, Viallon V, Claessens Y-E, Marqué S, et al. Temporal changes in management and outcome of septic shock in patients with malignancies in the intensive care unit. Crit Care Med. 2008;36(3):690–6. 18431262 10.1097/CCM.0B013E318165314B

[16] Australian and New Zealand Intensive Care Society Centre for Outcome and Resource Evaluation (ANZICS-CORE). Australian and New Zealand intensive care society adult patient database (ANZICS-APD) 2022. Available from: https://www.anzics.com.au/adult-patient-database-apd/

[17] Singer M, Deutschman CS, Seymour CW, Shankar-Hari M, Annane D, Bauer M, et al. The third international consensus definitions for sepsis and septic shock (sepsis-3). JAMA. 2016;315(8):801–10. 26903338 10.1001/jama.2016.0287 PMC4968574

[18] Paul E, Bailey M, Kasza J, Pilcher D. The ANZROD model: better benchmarking of ICU outcomes and detection of outliers. Crit Care Resusc. 2016;18(1):25–36. 26947413

[19] Knaus WA, Wagner DP, Draper EA, Zimmerman JE, Bergner M, Bastos PG, et al. The APACHE III prognostic system: risk prediction of hospital mortality for critically III hospitalized adults. Chest. 1991;100(6):1619–36. 1959406 10.1378/chest.100.6.1619

[20] Lambden S, Laterre PF, Levy MM, Francois B. The SOFA score—development, utility and challenges of accurate assessment in clinical trials. Crit Care. 2019;23(1):1–9. 31775846 10.1186/s13054-019-2663-7 PMC6880479

[21] Australian Government Department of Health and Aged Care. Monash modified model 2023. Available from: https://www.health.gov.au/topics/rural-health-workforce/classifications/mmm

[22] Azoulay E, Mokart D, Pène F, Lambert J, Kouatchet A, Mayaux J, et al. Outcomes of critically ill patients with hematologic malignancies: prospective multicenter data from France and Belgium—a groupe de recherche respiratoire en reanimation onco-hematologique study. J Clin Oncol. 2013;31(22):2810–8. 23752112 10.1200/JCO.2012.47.2365

[23] Liu J, Cheng Q, Yang Q, Li X, Shen X, Zhang L, et al. Prognosis-related factors in intensive care unit (ICU) patients with hematological malignancies: a retrospective cohort analysis in a Chinese population. Hematology. 2015;20(9):494–503. 25585045 10.1179/1607845414Y.0000000216

[24] Lemiale V, Pons S, Mirouse A, Tudesq J-J, Hourmant Y, Mokart D, et al. Sepsis and septic shock in patients with malignancies: a groupe de recherche respiratoire en réanimation onco-hématologique study. Crit Care Med. 2020;48(6):822–9. 32317596 10.1097/CCM.0000000000004322

[25] de Vries VA, Mueller MC, Arbous MS, Biemond BJ, Blijlevens NM, Kusadasi N, et al. Long-term outcome of patients with a hematologic malignancy and multiple organ failure admitted at the intensive care. Crit Care Med. 2019;47(2):e120–8. 30335623 10.1097/CCM.0000000000003526 PMC6336487

[26] Tan SC, Evans T, Durie ML, Secombe PJ, Pilcher D. Mortality among people admitted to Australian intensive care units for reasons other than COVID-19 during the COVID-19 pandemic: a retrospective cohort study. Med J Australia. 2023;218(10):467–73. 37080906 10.5694/mja2.51933

[27] Dang A, Thakker R, Li S, Hommel E, Mehta HB, Goodwin JS. Hospitalizations and mortality from non–SARS-CoV-2 causes among medicare beneficiaries at US Hospitals during the SARS-CoV-2 pandemic. JAMA Netw Open. 2022;5(3):e221754e. 35262712 10.1001/jamanetworkopen.2022.1754 PMC8908076

[28] Australian Government Department of Health and Aged Care. COVID-19 Australia epidemiology report 68. Commun Dis Intell 2022;46.

[29] Georges Q, Azoulay E, Mokart D, Soares M, Jeon K, Oeyen S, et al. Influence of neutropenia on mortality of critically ill cancer patients: results of a meta-analysis on individual data. Crit Care. 2018;22(1):1–10. 30514339 10.1186/s13054-018-2076-z PMC6280476

[30] Kim S-M, Kim Y-J, Kim Y-J, Kim W-Y. Prognostic impact of neutropenia in cancer patients with septic shock: a 2009–2017 nationwide cohort study. Cancers. 2022;14(15):3601. 35892860 10.3390/cancers14153601 PMC9332608

[31] Song J-U, Suh GY, Park HY, Lim SY, Han SG, Kang YR, et al. Early intervention on the outcomes in critically ill cancer patients admitted to intensive care units. Intensive Care Med. 2012;38(9):1505–13. 22592633 10.1007/s00134-012-2594-0

