# Supplementary material for: Sepsis mortality among patients with haematological malignancy admitted to intensive care 2000–2022: a binational cohort study
Source: Crit Care. 2024 May 6;28:148. doi: 10.1186/s13054-024-04932-0 (PMC11075186; doi:10.1186/s13054-024-04932-0)
Supplement: Supplementary file 1 — Additional file 1. Supplementary material. [file 13054_2024_4932_MOESM1_ESM.docx]

**Supplementary material**

**Figure S1: Inclusions and exclusions**


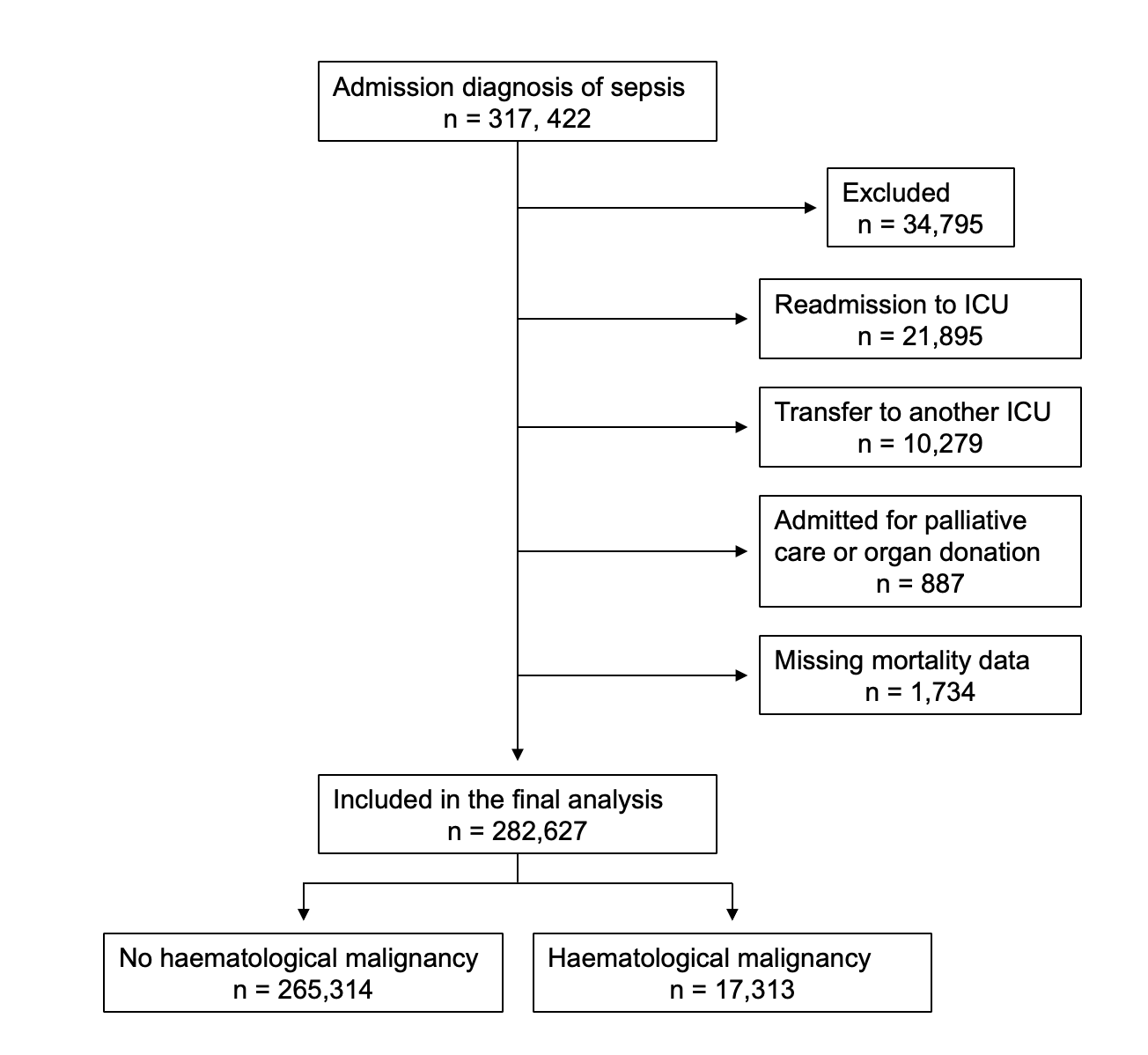


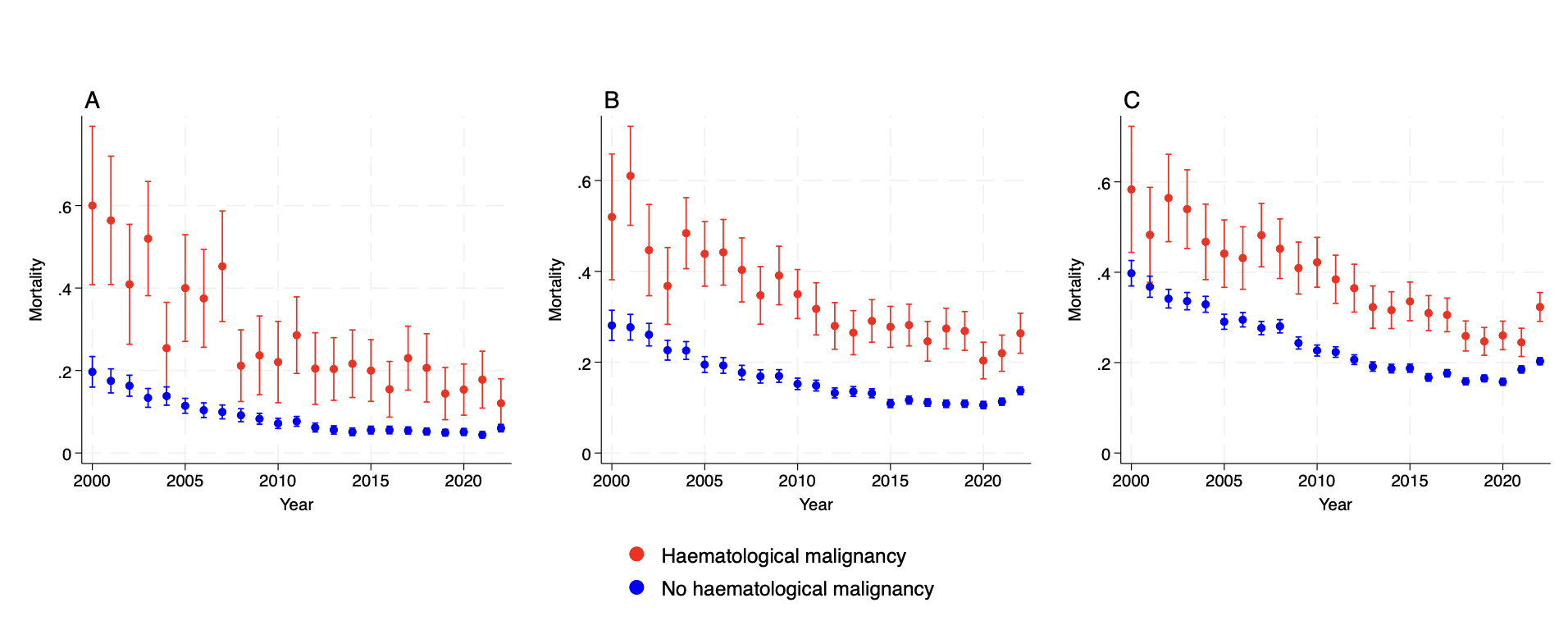


**Figure S2: Crude mortality in patients with and without haematological malignancy (A) Aged <45 years (B) Aged 45-64 years (C) Aged >= 65 years**

Error bars indicate 95% confidence interval

**Figure S3 Crude in-hospital mortality in patients with and without haematological malignancy across quartiles of Sequential Organ Failure Assessment score (SOFA)**

**(A) Quartile 1 (B) Quartile 2 (C) Quartile 3 (D) Quartile 4**

Error bars indicate 95% confidence interval


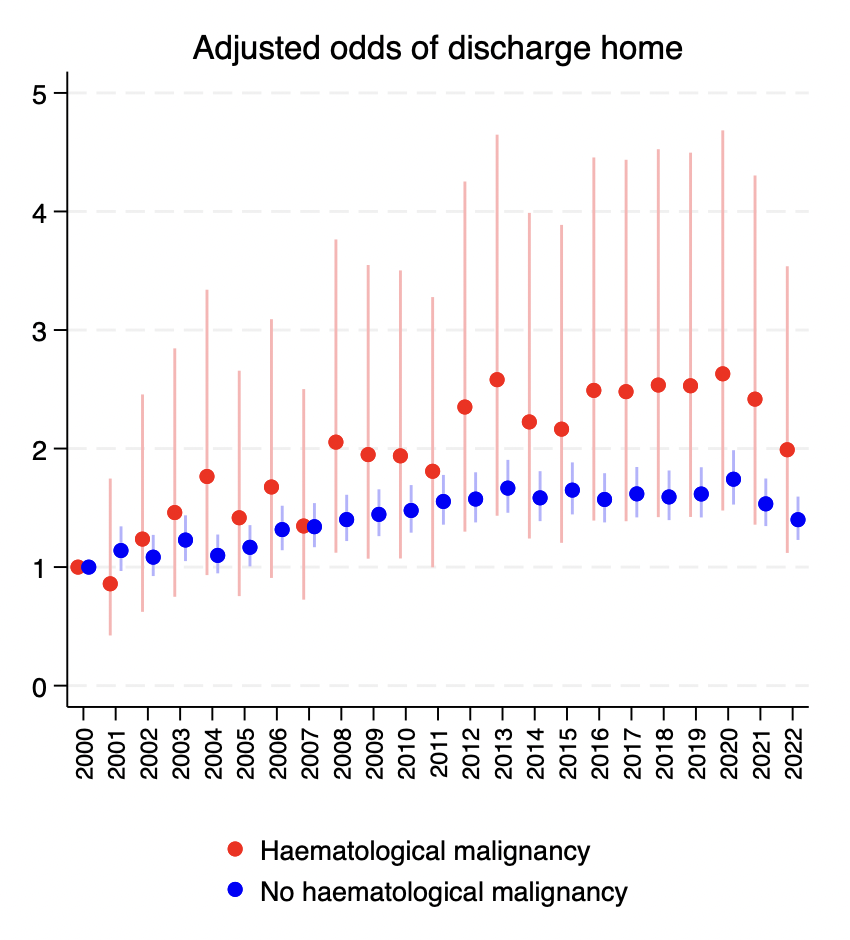


**Figure S4: Adjusted odds of discharge home by year in patients with and without haematological malignancy**

Results displayed as odds ratio +/- 95% confidence interval relative to the year 2000. Adjustments made for age, sex, presence of >/= 1 chronic comorbidity, SOFA score (quartile), post-operative status, mechanical ventilation, leukopenia and hospital site as a random effect.

**
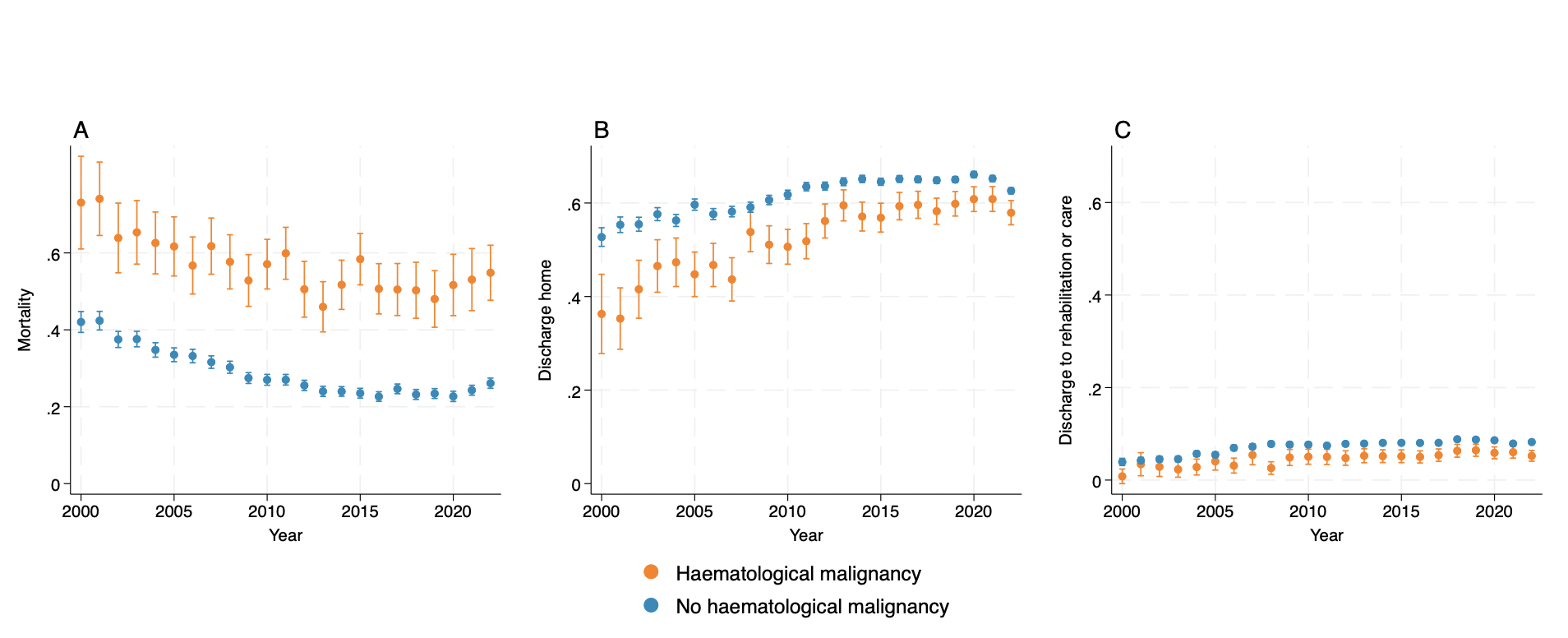
**

**Figure S5: Crude likelihood of hospital outcome in mechanically ventilated patients with and without haematological malignancy. (A) crude mortality (B) discharge home (C) discharge to rehabilitation or long-term care.**

Error bars indicate 95% confidence interval


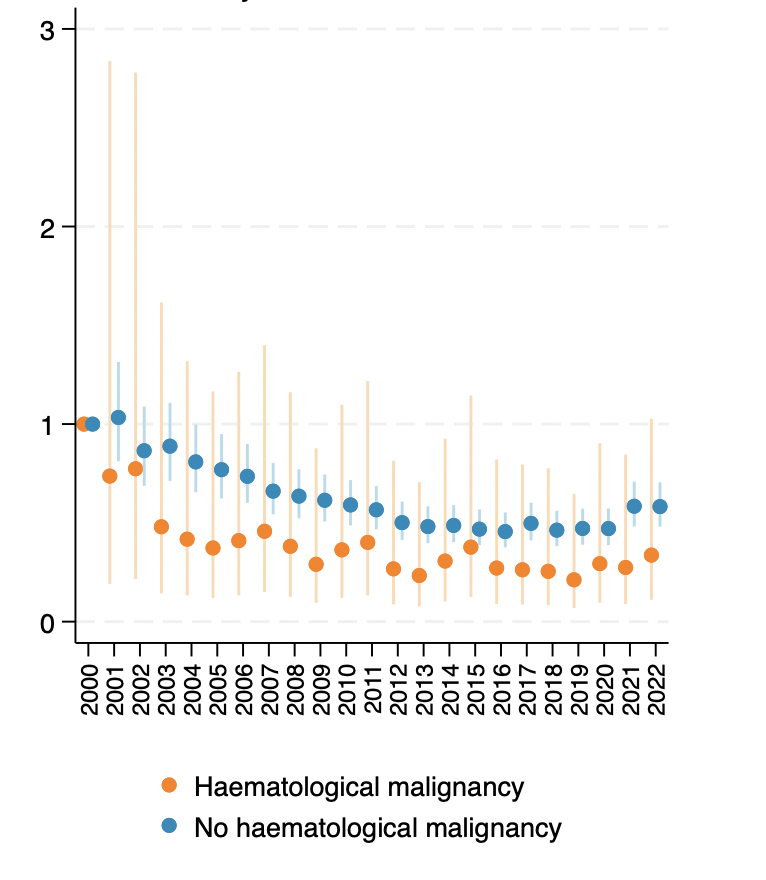


**Figure S6: Adjusted in-hospital mortality in patient with and without haematological malignancy who were mechanically ventilated**

Error bars indicate 95% confidence interval. Results displayed as odds ratio +/- 95% confidence interval relative to the year 2000. Adjustments made for age, sex, presence of >/= 1 chronic comorbidity, SOFA score, post-operative status, leukopenia and hospital site as a random effect.


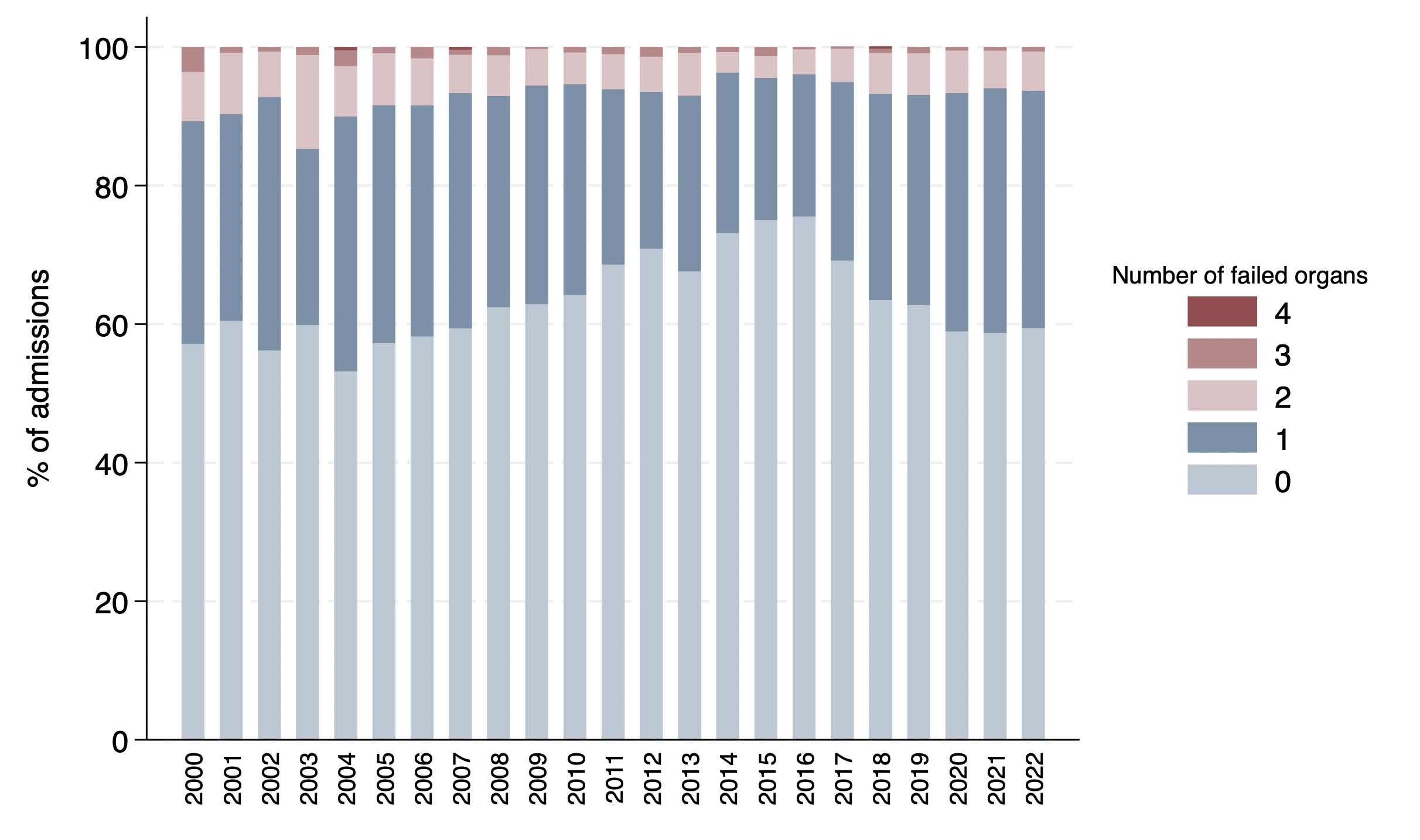


**Figure S7: Number of organ failures among patients with haematological malignancy and sepsis.**

Organ failures were renal, cardiovascular, respiratory, and hepatic. Organ failure was defined based on a SOFA sub-score of 3 or more as follows: Hepatic failure = bilirubin >101umol/L; respiratory failure = PaO_2_/FiO_2_ $\leq$ 200mmHg with requirement for ventilation or intubation; renal = creatinine of >299umol/L or oliguria <500ml/day; cardiovascular = mean arterial pressure < 60 mmHg and requirement for inotropes.


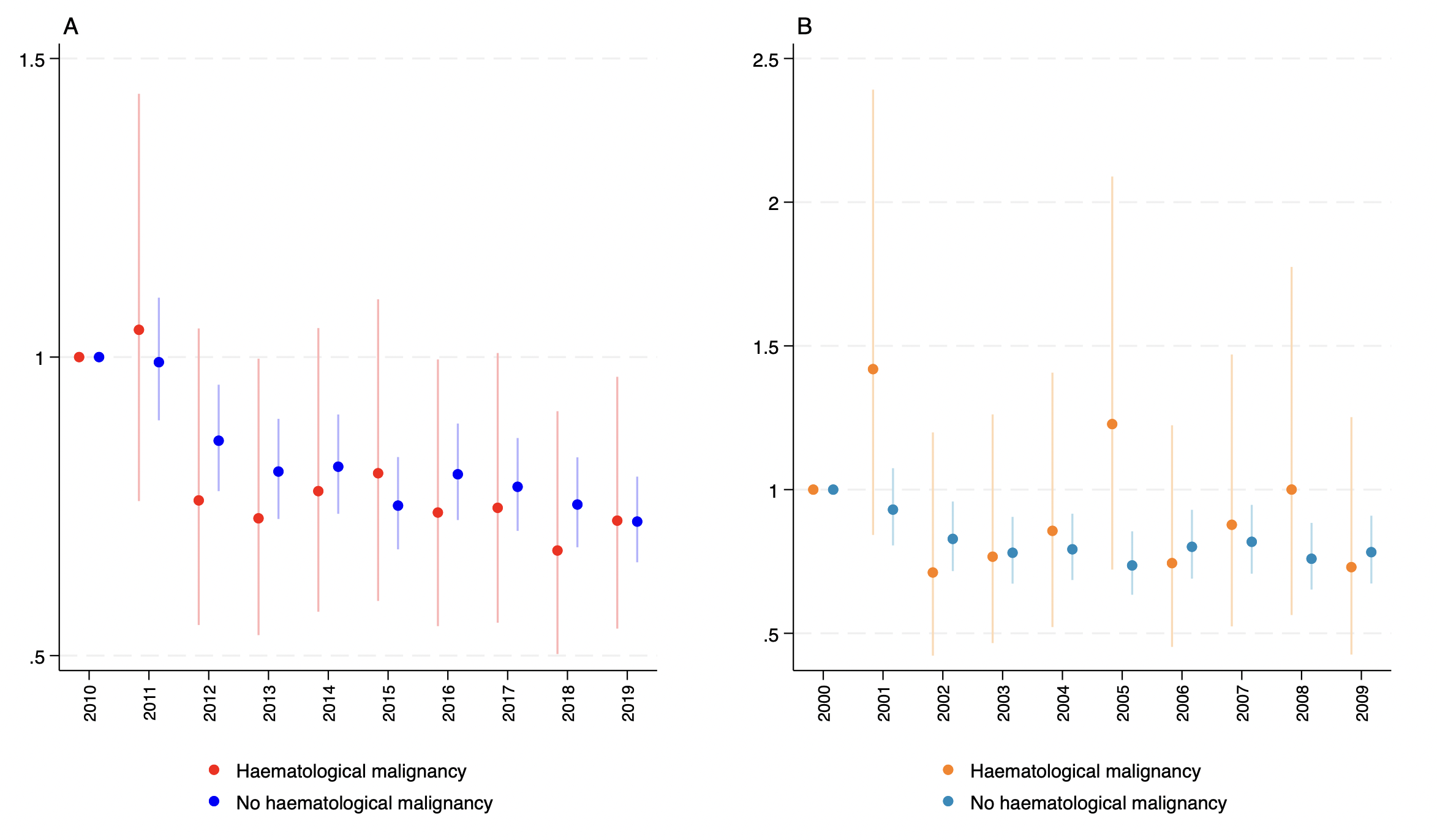


**Figure S8: Adjusted in-hospital mortality in patient with and without haematological malignancy, 2010-2019 (A) All sepsis (B) Mechanically ventilated patients with sepsis**

Error bars indicate 95% confidence interval. Results displayed as odds ratio +/- 95% confidence interval relative to the year 2010. Adjustments made for age, sex, presence of >/= 1 chronic comorbidity, SOFA score, post-operative status, leukopenia, and hospital site as a random effect
